# A Persistent Immune Response to an Acute Virus

**DOI:** 10.1371/journal.pmed.0020404

**Published:** 2005-11-01

Human parvovirus B19 (B19) can cause a wide range of conditions, which can depend to a large extent on an individual's immunologic and hematologic status. In a normal host, parvovirus infection can be asymptomatic or cause a range of clinical syndromes, from erythema infectiosum (“slapped cheek” disease) to chronic arthritis. Hydrops fetalis and fetal death are complications of intrauterine B19 infection, and patients who are immunocompromised or who have hematologic disorders are at risk of aplastic anemia.

To date, it has been believed that clearance of acute infection is associated with the lifelong emergence of antiviral IgG, but there is increasing evidence for an important role for cellular immune responses, which suggests there might be more to the way the body deals with this virus. One previous study detected CD8+ T cell responses—which kill virus-infected cells by cytokine secretion—in three asymptomatic seropositive individuals.

A better understanding of this aspect of the body's immune response could have important implications not only for vaccine development and treatment for B19, but also for other viral infections. The long-lasting CD8+ T lymphocyte response means that parvovirus-based vectors could be considered in vaccine strategies for other infections. The identification of B19 epitopes for CD8+ T lymphocytes also offers the chance to analyze the role of such effector cells in chronic arthritis, a disease in which B19 has been implicated. Better understanding of this response might also allow researchers to use B19 as a model for analyzing immunological memory, immunodominance, and the interplay between cellular and humoral immune responses to a common clinically relevant human pathogen.

In this month's *PLoS Medicine*, Adiba Isa and colleagues describe evolution of long-lived CD8+ immune responses against B19 in 11 adults with primary B19 infection. The phenotype of CD8+ T cells in acute B19 infection has not been studied before. Normally, the symptoms of this virus are short lived, but the immune responses showed here indicate sustained activity many months after initial infection. The team studied two groups of people: 11 who had been recently infected and five who had the virus many years ago. CD8+ T cell responses were mapped using a screening system, which took advantage of the B19's compact and stable viral genome. The researchers used human leukocyte antigen (HLA)–peptide multimeric complexes to detect CD8+ T cell responses during acute B19 infection.

The researchers believe their results show a new style of host–virus relationship in which an acute human viral infection induces persistent activated CD8+ T cell responses. They found that these responses continued to increase, in some cases for many months, long after acute symptoms had resolved—something not seen with other viruses. For example, responses to HIV are strong in acute infection but typically decline as the virus is controlled.

Alongside the expansion of antiviral responses was the continued change in the B19 CD8+ T cells, as indicated by a range of markers. The evolution of markers could represent a maturation pathway, said the authors, driven by restimulation in vivo with antigen.

This T cell response to B19 infection indicated the persistence of antigen long after the resolution of acute infection. However, the authors said the status of the virus postinfection is still not understood. For example, in this study, PCR analysis found B19 DNA in the blood early on during infection, but assays were negative after 6–12 months when T cell populations remained active. The most likely reason for these immune responses is low-level replication at a tissue site for weeks or months after infection, suggest the authors. But this hypothesis can only be checked by more sensitive PCR assays.

In addition, the relationship between joint or bone marrow pathology and T cell responses seen was not clear. In the patients with arthritis, the most active CD8+ T cell responses were seen at stages where joint symptoms had resolved.

Altogether, this study is the first demonstration that a virus, not considered a true or classical persistent infection, can lead to a persistent activated CD8+ T cell response. It suggests that B19 persists after acute infection, provoking sustained activated CD8+ T cell responses, which might then play a role in viral clearance. A better understanding of cytotoxic T lymphocytes could improve understanding of the role of T cells in acute and persistent infections and be of great value in vaccine design and immunotherapy.

